# Molecular and clinical characterization of TMEM71 expression at the transcriptional level in glioma

**DOI:** 10.1111/cns.13137

**Published:** 2019-06-10

**Authors:** Kuan‐Yu Wang, Ruo‐Yu Huang, Xue‐Zhi Tong, Ke‐Nan Zhang, Yan‐Wei Liu, Fan Zeng, Hui‐Min Hu, Tao Jiang

**Affiliations:** ^1^ Department of Neurosurgery, Beijing Neurosurgical Institute Capital Medical University Beijing China; ^2^ Chinese Glioma Cooperative Group (CGCG) Beijing China; ^3^ Department of Neurosurgery, Beijing Tiantan Hospital Capital Medical University Beijing China; ^4^ Department of Radiotherapy, Beijing Tiantan Hospital Capital Medical University Beijing China; ^5^ Department of Neuropathology, Beijing Neurosurgical Institute Capital Medical University Beijing China

**Keywords:** chemoresistance, glioblastoma multiforme, glioma, glioma stem cells, immune response, TMEM71

## Abstract

**Background:**

Glioma is the most common and aggressive type of primary brain tumor in adults. Although radiotherapy and chemotherapy are used in the treatment of glioma, survival remains unsatisfactory. Chemoresistance is one of the primary reasons for the poor prognosis of glioma. Several studies have demonstrated that glioma stem cells (GSC) may be one of the reasons for chemoresistance. In this article, we attempt to search for a new biomarker related to GSC and chemoresistance in glioma.

**Methods:**

We used three datasets (GSE23806, COSMIC, and CGGA) to search for the genes related to GSC, temozolomide (TMZ) resistance, and overall survival. The selected gene was investigated with respect to the relationship between mRNA levels and clinical characteristics in the CGGA and TCGA dataset. Gene ontology (GO) analysis was used for bioinformatics analysis. Kaplan‐Meier survival analysis and Cox regression analysis were used for survival analysis.

**Results:**

The transmembrane protein 71 (TMEM71) gene was selected for further research. TMEM71 was highly expressed in GSCs and TMZ‐resistant cells. The TMEM71 mRNA levels increased with increasing grades of glioma. In IDH‐wild‐type and MGMT‐unmethylated samples, TMEM71 was overexpressed. The TMEM71 transcript levels were also increased significantly in mesenchymal subtype gliomas. GO analysis demonstrated that TMEM71 was related to the immune and inflammatory responses, cell proliferation, cell migration, chemotaxis, and the response to drugs. Specifically, PD‐1, PD‐L1, TIM‐3, and B7‐H3 were tightly associated with TMEM71 expression. This result indicates that TMEM71 may play an important role in the immune response. More importantly, high expression of TMEM71 was correlated with short survival time in both glioma and glioblastoma patients.

**Conclusion:**

In summary, TMEM71 expression was increased in GBM and associated with immune response. Our study suggests that TMEM71 may function as an oncogene and serve as a new effective therapeutic target for the treatment of glioma.

## 1 INTRODUCTION

Glioma is the most common and aggressive type of primary brain tumor in adults.^1^, ^2^ Current therapies including surgery, chemotherapy, and radiotherapy are partly effective for glioma. Therefore, treatment for glioma remains a serious clinical and scientific problem. The median survival in patients with glioma remains unsatisfactory, especially in glioblastoma multiforme (GBM), which is the most malignant type.^1^, ^3^ For further understanding the molecular mechanisms behind the disease manifestation and progression, a new prognostic biomarker and effective therapeutic targets for GBM still need to be identified.

Recently, the most common drug used in chemotherapy for glioma is temozolomide (TMZ), which is a standard treatment.^1^ Although TMZ has improved both the overall survival (OS) and the progression‐free survival (PFS), patients have often suffered from chemoresistance after using TMZ. The mechanism of the chemoresistance is still unclear. Several studies focus on the glioma stem cells (GSC), which may be related to chemoresistance.^4^

Cancer stem cells (CSC) are a subpopulation of tumor tissue and are postulated as a mediator of chemoresistance. After chemotherapy, surviving CSC may later differentiate into rapidly proliferating tumor cells and lead to tumor recurrence.^5^ In recent years, multiple genes have been discovered as signatures of CSC. CD133 is one of the most studied CSC biomarkers.^6^ CD133‐positive glioma cells can grow spheres in serum‐free medium, whereas CD133‐negative cells cannot grow spheres.^7^, ^8^ Several studies have demonstrated that knocking down CD133 could weaken tumorigenicity.^8^ SOX2 is another signature of CSC. SOX2 is highly expressed in CSC, and it can promote the self‐renewal of CSC.^9^ Moreover, other CSC‐relevant molecular markers, such as CD15, CD36, A2B5, L1CAM, and CD44, have been reported by researchers.^10^, ^11^, ^12^, ^13^

Recently, there has been insufficient research on utilizing GSC‐related genes to predict prognosis and chemoresistance. For this reason, we focused on these genes to search for a new biomarker in glioma. In the present study, we discovered that transmembrane protein 71 (TMEM71) mRNA expression was related to TMZ chemoresistance, and high expression of this protein was associated with GSC. Further research revealed that TMEM71 expression was significantly higher in GBM than in lower‐grade glioma tissues. The TMEM71 expression level was also positively related to the histological grades of gliomas. The patients with high TMEM71 expression level tend to enrich in IDH wild‐type and MGMT unmethylated samples. Bioinformatics analysis indicates that overexpression of TMEM71 was involved in multiple crucial biological processes including immune and inflammatory response, cell proliferation, cell migration, response to drug, and so on. Collectively, these results suggest that TMEM71 is a potential oncogene in GBM and that it may serve as a therapeutic target for this disease.

## 2 METHODS

### 2.1 Samples

In this article, eight datasets were used for the analysis. The Chinese Genome Atlas (CGGA) RNA sequencing dataset includes 325 samples of glioma RNA sequencing data, and clinical information was obtained from the publicly available website (http://www.cgga.org.cn). The CGGA RNA microarray dataset includes 301 samples. The Cancer Genome Atlas (TCGA) RNA sequencing dataset and the RNA microarray dataset were downloaded from The Cancer Genome Atlas (TCGA) (http://cancergenome.nih.gov). Other two datasets were obtained from The Repository for Molecular Brain Neoplasia Data (REMBRANDT, http://caintegrator-info.nci.nih.gov/REMBRANDT) and the GSE16011 microarray database (https://www.ncbi.nlm.nih.gov/geo/). In the datasets, only samples with definite WHO classification were used for further analysis. The GSE23806 database was downloaded from the GEO website (https://www.ncbi.nlm.nih.gov/geo/), and it includes the stem cell RNA microarray data. The COSMIC Cell Lines Project (https://cancer.sanger.ac.uk/cell_lines/) was obtained from the public website for the drug sensitivity analysis.

### 2.2 Bioinformatics analysis

Gene ontology (GO) was used to analyze biological function.^14^ The correlation between TMEM71 mRNA expression and other genes was analyzed by the Pearson correlation analysis using the R programming language. The positive correlative gene (*r* > 0.4, *P* < 0.05) and the negative correlative gene (*r *< −0.4, *P* < 0.05) were chosen for analysis by DAVID (http://david.abcc.ncifcrf.gov/home.jsp) to detect the biological processes that were correlated with TMEM71 expression. The results were showed by heatmap using R programming language.

### 2.3 Statistical analysis

In this study, SPSS 16.0 (Armonk, NY, USA), the R programming language 3.2.5 and the GraphPad Prism 7.0 statistical software (La Jolla, CA, USA) were used to conduct the analysis and generate the graph. Student's *t* test was used to compare the different expression levels between grades or subtypes. KaplanssMeier survival analysis and univariate and multivariate Cox regression analysis were used for the survival analysis. The pheatmap, pROC, circcos, and corrgram^15^, ^16^ were generated by several R packages. *P* value < 0.05 was considered statistically significant.

## 3 RESULTS

### 3.1 Gene selection

**Figure 1:**
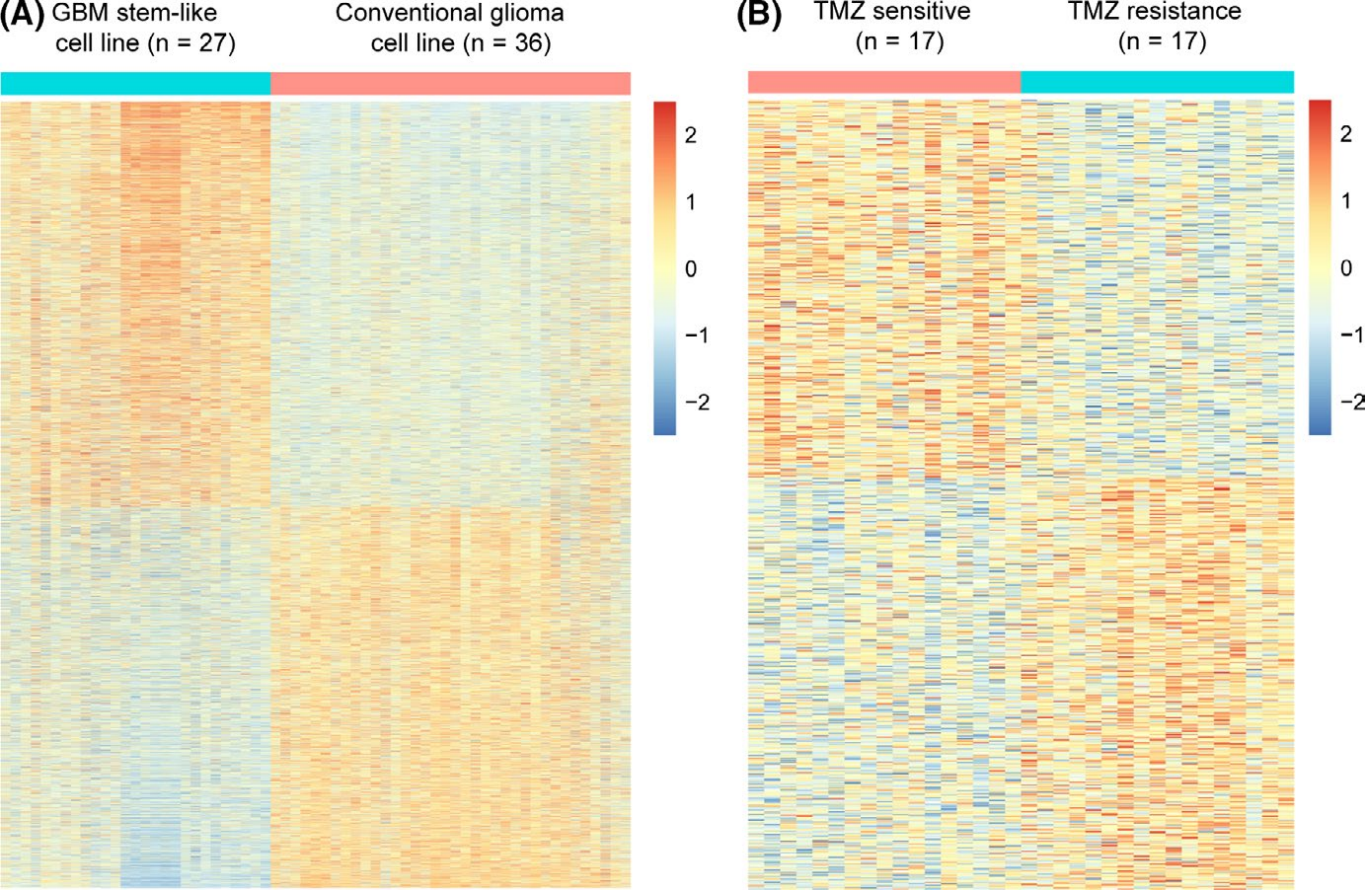
(A) The different genes between GSCs and conventional GBM cell lines. (B) The different genes between TMZ‐sensitive cell lines and TMZ‐resistant cell lines

Student's *t* test was conducted in the GSE23806 dataset to identify genes with different expressions between conventional glioma cell lines (n = 36) and glioblastoma stem‐like cell lines (n = 27) (*P* < 0.05). The detailed information of the cell lines was given by the dataset to choose which type the cell line was. In the glioblastoma stem‐like cell line, 6752 genes exhibited high expression, and they were selected for further analysis (Figure 1A). In COSMIC dataset, the half maximal inhibitory concentration (IC50) of TMZ was given by the dataset and the data of glioma cell lines was chosen to do the analysis. According to the median IC50 of TMZ to the glioma cell lines, the cell lines were divided into two groups. Student's *t* test was also used in the COSMIC dataset to identify genes with different expression between different sensitivities to TMZ according to the IC50. In the high‐IC50 group, 447 genes were highly expressed and were selected for further analysis (*P* < 0.05, Figure 1B). Next, we used univariate Cox regression in the CGGA RNA sequencing dataset to analyze the genes related to survival (*P* < 0.05) in GBM samples (n = 144). The genes significantly related to survival were retained (n = 1302). Only three genes were left in all three datasets: TMEM71, CA12, and FUT7 (Supplementary Figure S1A). We focus on TMEM71, which has been hardly researched in glioma, even in tumors (Supplementary Figure S1B, C).

### 3.2 Heterogeneity of TMEM71 expression in glioma

**Figure 2:**
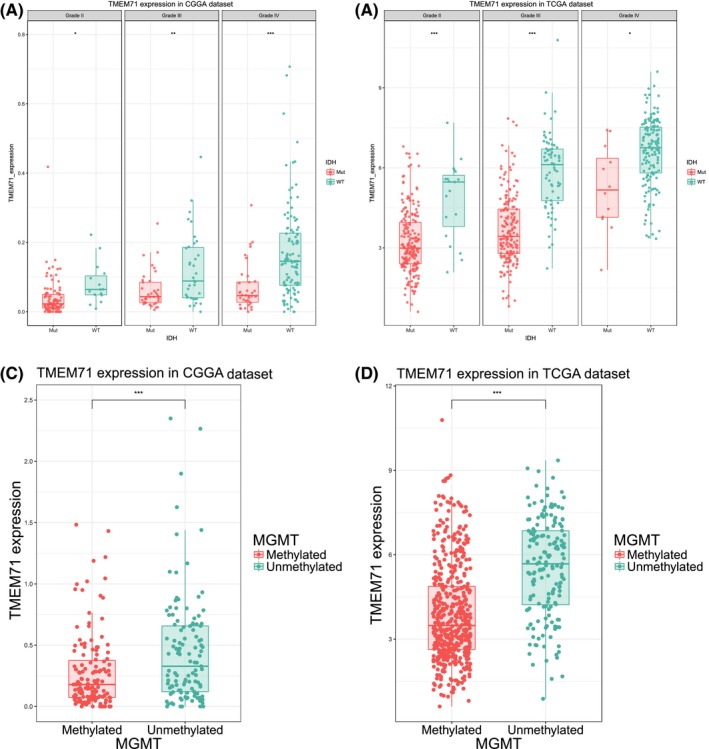
(A‐B) TMEM71 expression in different grades according to IDH status from the CGGA and TCGA databases. (C‐D) TMEM71 expression according to MGMT status from the CGGA and TCGA databases. ***P < 0.001, **P < 0.01, *P < 0.05

By examining the RNA sequencing data of glioma from the CGGA database, we found that the expression level of TMEM71 was positively correlated with tumor grade. Compared with lower‐grade gliomas, WHO grade IV gliomas (glioblastoma) showed a higher TMEM71 expression in the CGGA database (Figure 2A). This result suggests that TMEM71 overexpression plays a key role in the progression of glioma, consistent with other TMEM family members in various types of malignant tumors, as reported previously. According to previous studies, glioma patients with IDH mutation and MGMT methylation generally exhibit a better prognosis. Thus, we further evaluated the relationship between TMEM71 expression level and IDH mutation and MGMT methylation status. The results showed that TMEM71 was highly enriched in IDH‐wild‐type glioma and MGMT‐unmethylated glioma (Figure 2A, C). This finding indicates that the overexpression of TMEM71 was associated with the malignant progression of glioma. The results mentioned above were validated in the TCGA RNA sequencing dataset (Figure 2B, D), REMBRANDT RNA microarray dataset (Supplementary Figure S2A), and GSE16011 RNA microarray dataset (Supplementary Figure S2B).

### 3.3 TMEM71 expression level shows a subtype preference

**Figure 3:**
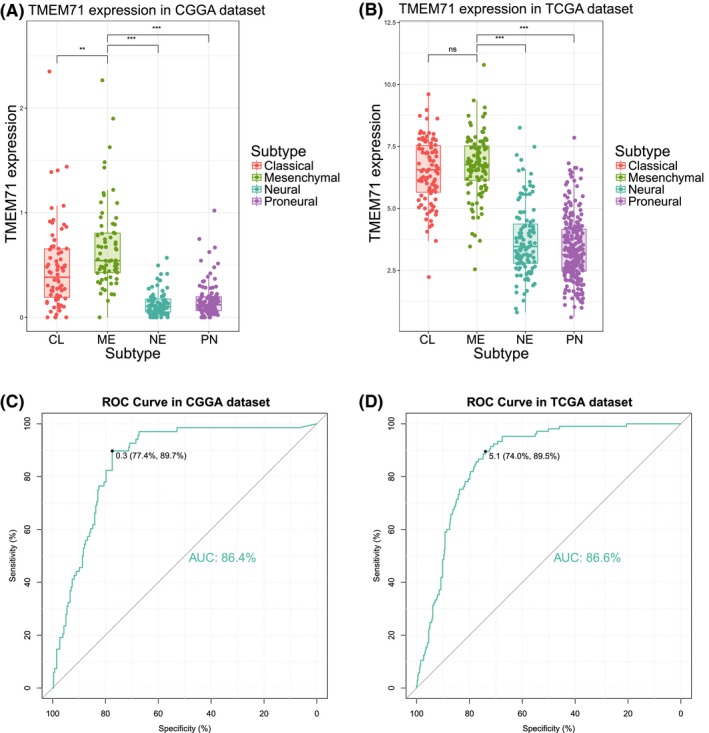
(A‐B) TMEM71 expression in molecular subtypes from the CGGA and TCGA datasets. (C‐D) TMEM71 expression was used to predict mesenchymal subtype or not in both CGGA and TCGA subtype. The AUC was more than 80% in both datasets. ***P < 0.001, **P < 0.01, *P < 0.05

To further explore the molecular relevance between TMEM71 and glioma, we analyzed TMEM71 expression in different molecular subtypes. The results showed that the TMEM71 expression levels in the four molecular subtypes were considerably different in the CGGA and TCGA datasets. Compared with the other three subtypes, TMEM71 was significantly upregulated in the mesenchymal subtype, which is typically associated with poor outcomes (Figure 3A, B and Supplementary Figure S2C, D). Moreover, ROC curves were generated for TMEM71 expression and the mesenchymal subtype. As shown in Figure 3C and D, the area under the curve (AUC) was up to 86.4% and 86.6% in the CGGA and TCGA sequencing datasets, respectively. These results enlightened us that TMEM71 was a potential biomarker for mesenchymal subtype glioma.

### 3.4 TMEM71‐related GSC markers

**Figure 4:**
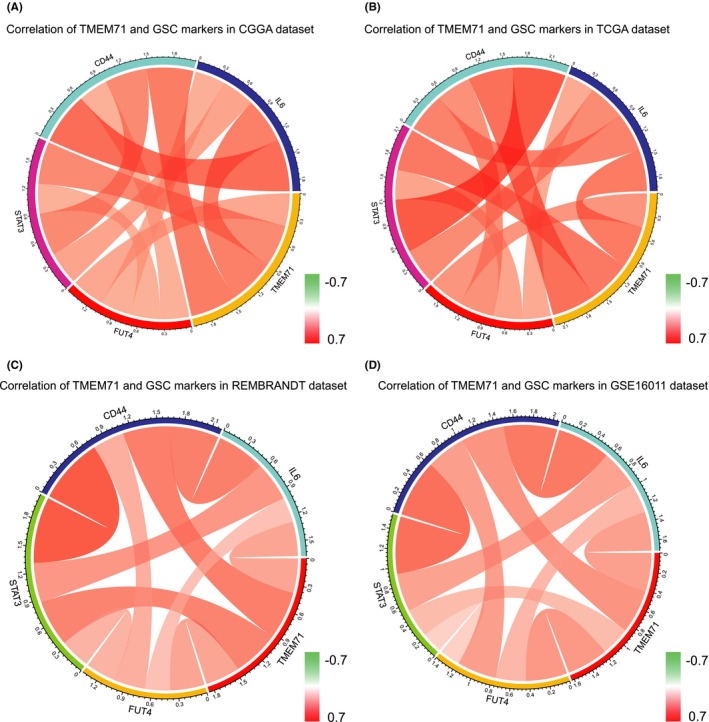
(A‐B) The relation between TMEM71 expression and GSC markers expression in the CGGA and TCGA databases. (C‐D) The relation between TMEM71 expression and GSC markers expression in the REMBRANDT and GSE16011 databases

In our research, we found that TMEM71 was upregulated in GSCs. To conduct further research on TMEM71‐related GSC markers, we chose several genes that were related to GSC according to previous reports.^17^ Pearson correlation analysis was used to search for the GSC‐related genes that correlated with TMEM71 expression. In both CGGA and TCGA datasets, it was found that IL6, STAT3, CD44, and FUT4 were positively related to TMEM71 expression (Figure 4A, B). The similar results were also obtained in REMBRANDT and GSE16011 datasets (Figure 4C, D). Previous research had demonstrated that hyperactivation of the IL6/STAT3 pathway was required for GSC self‐renewal and tumorigenesis.^18^, ^19^ TMEM71 may play an important role in the pathway.

### 3.5 TMEM71 related biological process

**Figure 5:**
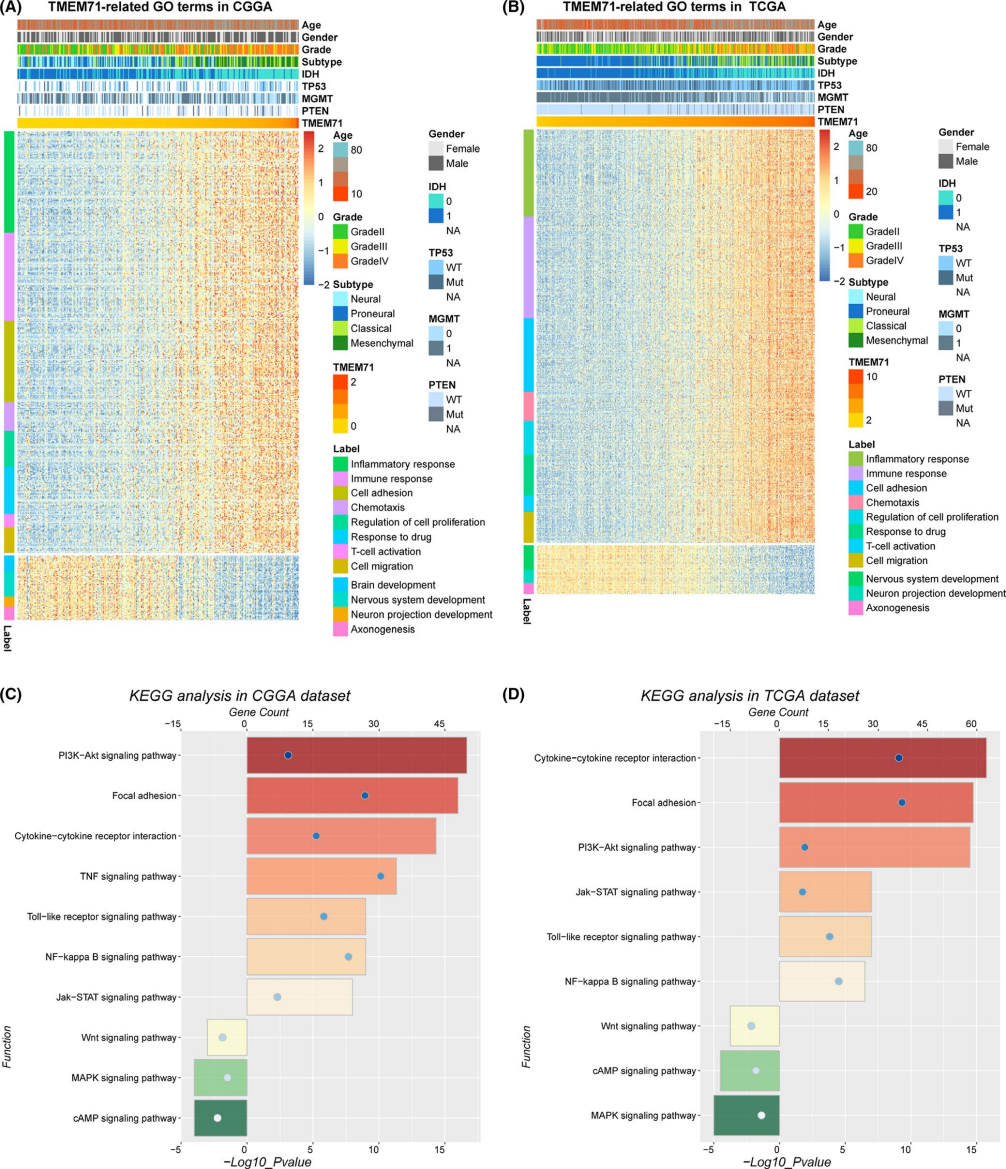
Biological function and pathway analysis in CGGA and TCGA datasets. (A‐B) Gene ontology analysis of TMEM71 expression in glioma. The samples were ranked according to TMEM71 expression from low to high. (C‐D) KEGG analysis of TMEM71 expression in glioma. The bar charts represented the count and the circle represented the P value. The color also represented the count

Based on the above findings, we inferred that TMEM71 may exhibit essential biologic functions in glioma. To further investigate the biological process tightly associated with TMEM71 expression, Pearson correlation analysis was performed to identify the genes that tightly correlated with TMEM71 expression (Pearson |*R*| > 0.4) in the CGGA and TCGA sequencing datasets. Significantly related genes were used for gene ontology (GO) analysis with DAVID. According to the results shown in Figure 5A and B, genes that positively correlated with TMEM71 expression were highly enriched in the immune and inflammatory response, cell proliferation and migration, T‐cell activation, chemotaxis, and response to drug in GO terms. Genes that negatively correlated with TMEM71 tended to be enriched in biological processes that are normal and indispensable, such as the nervous system development and neuron projection development (Supplementary Figure S3A, B). The KEGG pathway analysis revealed that TMEM71 expression is positively related to the PI3K‐AKT signaling pathway and the JAK‐STAT signaling pathway and negatively related to the Wnt and cAMP signaling pathway (Figure 5C, D). All the results mentioned above were shared by the two datasets. These analyses indicate that TMEM71 might play a key role in the malignant progression of glioma.

### 3.6 TMEM71 was synergistic with immune checkpoint members in tumor‐induced immune response

**Figure 6:**
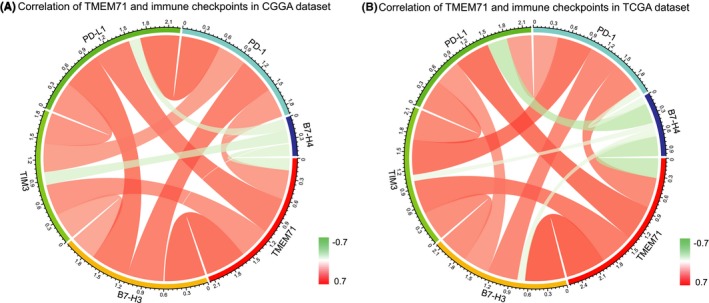
(A‐B) Correlation between TMEM71 and immune checkpoints in the CGGA and TCGA databases

Several immune checkpoint members were enrolled in the analysis and assessed as therapeutic targets, such as PD‐1, PD‐L1, B7‐H3, B7‐H4, and TIM‐3.^20^, ^21^, ^22^ Pearson correlation analysis was used to analyze the relation between TMEM71 and these five factors in both CGGA and TCGA datasets. In both datasets, TMEM71 showed a high positive correlation with PD‐1 and PD‐L1, indicating its association with the PD‐1/PD‐L1 pathway (Figure 6A, B). TIM‐3 and B7‐H3 were also tightly associated with TMEM71 expression (Figure 6A, B). This result indicates that TMEM71 may play an important role in immune response.

### 3.7 TMEM71‐related inflammatory activities

**Figure 7:**
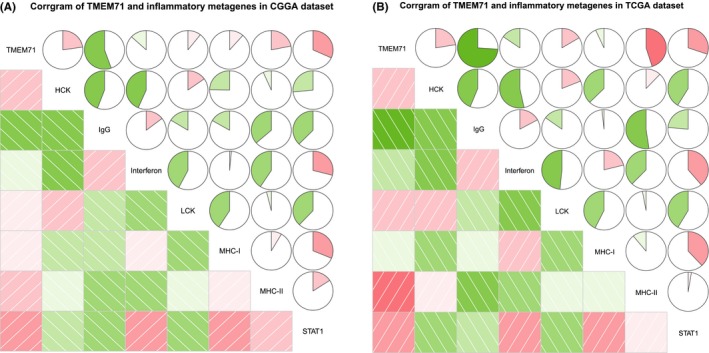
(A‐B) The relation between TMEM71 and immune response in the CGGA and TCGA databases

To conduct further research on TMEM71‐related inflammatory activities, we chose seven clusters that represented several types of inflammation and immune responses. These seven clusters were calculated by Gene Sets Variation Analysis (GSVA) to generate seven metagenes.^23^ In both CGGA and TCGA datasets, it was revealed that TMEM71 expression was positively related to HCK, LCK, MHC‐II, and STAT1 while negatively related to IgG and interferon (Figure 7A, B). These results suggest that TMEM71 expression was upregulated with the activation of macrophages and T‐cell signaling transduction.

### 3.8 TMEM71 was determined to be an independent prognostic factor for GBM patients

**Figure 8:**
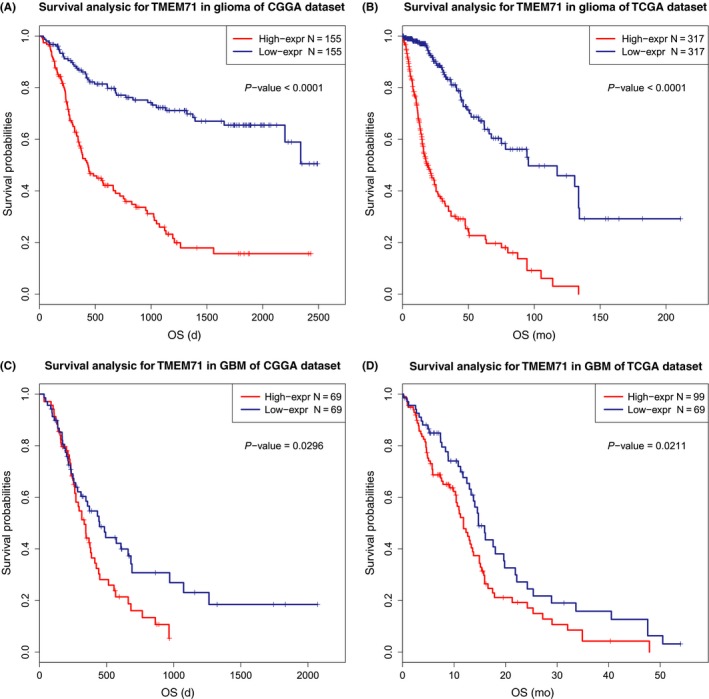
(A‐B) Survival analysis in glioma from the CGGA and TCGA databases. (C‐D) Survival analysis in GBM from the CGGA and TCGA databases

To evaluate the prognostic value of TMEM71 expression in glioma patients, Kaplan‐Meier (K‐M) survival curve analysis was performed with the data from the CGGA and TCGA RNA sequencing datasets. The results showed that the overall survival (OS) time of patients with higher TMEM71 expression in glioma is shorter than patients with lower TMEM71 expression (Figure 8A, B). Considering the significant biological heterogeneity between GBM and lower‐grade glioma, we additionally analyzed the prognostic value of TMEM71 in GBM patients from the two datasets. We found that patients with high TMEM71 expression exhibited a significantly shorter survival than those with lower TMEM71 expression (Figure 8C, D). Similar results were observed in the TCGA microarray and REMBRANDT datasets (Supplementary Figure S4A, B). The ROC curve analysis was performed to validate the prognostic value of TMEM71. Compared with age at diagnosis and tumor grade, the AUCs for TMEM71 expression level in the prediction of 3 years of survival in glioma were close to “grade” and higher than “age” (Supplementary Figure S4C, D). These results revealed that TMEM71 is a negative prognostic factor in glioma and GBM patients.

Univariate Cox regression analysis was performed to further estimate the prognostic value of TMEM71 in GBM patients in the CGGA dataset. The results showed that high TMEM71 expression, Karnofsky Performance Status (KPS) score, MGMT methylation state, radiotherapy, and chemotherapy after resection were shown to be significantly associated with OS of GBM patients (Table 1). Thus, we performed a multivariate Cox regression analysis, and the result revealed that the TMEM71 expression is an independent prognostic biomarker for GBM patients (Table 1). Furthermore, we obtained similar results for the TCGA microarray and RNA sequencing datasets (Tables S1andS2). Those results implied that TMEM71 might be a novel independent prognostic biomarker for GBM patients.

**Table 1:** Univariate and multivariate analysis of OS in CGGA RNA sequencing dataset, GBM

Variables	Univariate analysis		Multivariate analysis	
	HR (95% CI)	*P* value	HR (95% CI)	*P* value
TMEM71 expression	7.035 (1.571‐31.494)	0.011	18.43 (2.463‐138.02)	0.005
Age at diagnosis	1.005 (0.988‐1.022)	0.569	–	–
Gender	1.227 (0.795‐1.893)	0.355	–	–
TCGA subtype	1.082 (0.900‐1.301)	0.403	–	–
IDH mutation status	0.685 (0.406‐1.157)	0.157	–	–
MGMT methylation	0.564 (0.364‐0.872)	0.01	0.921 (0.506‐1.673)	0.786
Radiotherapy	0.412 (0.259‐0.654)	<0.001	0.498 (0.274‐0.907)	0.023
Chemotherapy	0.336 (0.214‐0.528)	<0.001	0.442 (0.251‐0.778)	0.005
KPS	0.970 (0.955‐0.986)	<0.001	0.961 (0.942‐0.981)	<0.001

## 4 DISCUSSION

GBM contributes to approximately 50% gliomas. Despite the combination of precise surgery, chemotherapy, and radiotherapy, the prognosis for patients with GBM remains dismal.^1^, ^2^ For decades, there has been no breakthrough in the treatment of GBM, besides temozolomide. Thus, there is an urgent need for novel therapeutic approaches. The origination and development of GBM is a complex and multistep process that is affected by various regulators. Therefore, identifying these regulators is a feasible approach.

Transmembrane protein 71 (TMEM71) belongs to a large family of genes encoding transmembrane (TMEM) proteins. TMEM family members have been reported to be involved in many physiological processes such as forming the plasma membrane ion channels, activating signal transduction pathways, mediating cell chemotaxis, adhesion, apoptosis, autophagy, and so on.^24^, ^25^ In recent years, the involvement of TMEMs in malignancy has stimulated interest among researchers. For instance, TMEM14A participates in ovarian carcinogenesis and metastasis.^26^ TMEM88 has been reported to be associated with platinum resistance in ovarian cancer and stimulate invasion and metastasis in breast cancer by interacting with disheveled (Dvl) proteins.^27^, ^28^ TMEM45A mediates proliferation and invasion of glioma cells and protects breast and liver cancer cells from drug‐induced apoptosis,^29^ and TMEM45B promotes the proliferation, migration, and invasion of gastric cancer cells via the JAK2/STAT3 signaling pathway.^30^ TMEM205 is tightly associated with cisplatin resistance.^31^, ^32^ However, the function and expression characteristics of TMEM71, a member of TMEMs, have never been reported in any type of malignant tumor.

In the present study, our results indicate that TMEM71 may have an oncogenic effect on GBM. TMEM71 expression was upregulated in GSCs and TMZ‐resistant cells. We analyzed the TMEM71 expression in the RNA‐seq data from the CGGA and TCGA datasets. It was determined that TMEM71 expression was significantly upregulated in GBM samples than in lower‐grade samples. Moreover, the results showed that high expression of TMEM71 was highly enriched in the IDH‐wild‐type, MGMT unmethylation, and mesenchymal‐phenotype gliomas. As is well known, MGMT unmethylation indicated that the patient was insensitive to TMZ treatment.^1^ It coincided with the observation that patients with TMEM71 overexpression were insensitive to TMZ treatment. Meanwhile, these molecular characteristics generally led to poor clinical outcome. All these results indicate that TMEM71 expression was tightly associated with the development and malignancy progression of glioma. This indicates that revealing the mechanism of TMEM71 in glioma is essential for the treatment of this deadly disease.

In many researches, it had been verified that the existence of GSCs was a mediator of chemoresistance. GSCs might survive after chemotherapy and differentiate into tumor cells and lead to tumor recurrence.^5^ In our research, it was demonstrated that TMEM71 expression was upregulated in GSCs. Meanwhile, it was found that TMEM71 expression was positively related with IL6, STAT3, CD44, and FUT4 which were reported as classical markers to GSCs.^17^ Several researches had demonstrated that the IL6/STAT3 pathway was related to GSCs tumorigenesis and self‐renewal.^18^, ^19^ In the present research, our results indicated that TMEM71 may play an important role in IL6/STAT3 pathway. It indicated that relevant target therapy could be used to block this signaling pathway which was related to stem cell growth. This would be a new direction of glioma treatment.

Next, we performed a series of bioinformatics analysis. As shown in Figures, TMEM71 was tightly associated with the immune and inflammatory response, cell proliferation, and cell migration, which are key steps for tumor progression. Our research demonstrated that the expression of TMEM71 was closely correlated with the inflammatory and immune responses, which acted as a higher invasion of immune cells. These results indicate that TMEM71 may inhibit the immune response. Thus, it can be inferred that anti‐TMEM71 may serve as an effective treatment.

Moreover, the expression of TMEM71 was positively related to chemotaxis and the response to drugs. This might be the reason that TMEM71 overexpression leads to chemoresistance to TMZ therapy. Nevertheless, we know little regarding the function of TMEM71 in GBM. It is necessary to conduct further research.

## 5 CONCLUSIONS

In conclusion, the results described above revealed that TMEM71 acts as an oncogene in GBM. It may be related to GSC, TMZ resistance, and immune response. The upregulation of TMEM71 may indicate poor sensitivity to TMZ therapy. The study also points to TMEM71 as a potential therapeutic target for the clinical management of GBM.

## ETHICAL APPROVAL AND CONSENT TO PARTICIPATE

This study was approved by the Beijing Tiantan Hospital institutional review board (IRB), and informed consent was obtained from all individual participants who were included in this study.

## CONFLICT OF INTEREST

The authors declare no conflict of interest.
