# Supplementary figures and images for: Molecular and clinical characterization of TMEM71 expression at the transcriptional level in glioma
Source: CNS Neurosci Ther. 2019 Jun 10;25(9):965–75. doi: 10.1111/cns.13137 (PMC6698980; doi:10.1111/cns.13137)

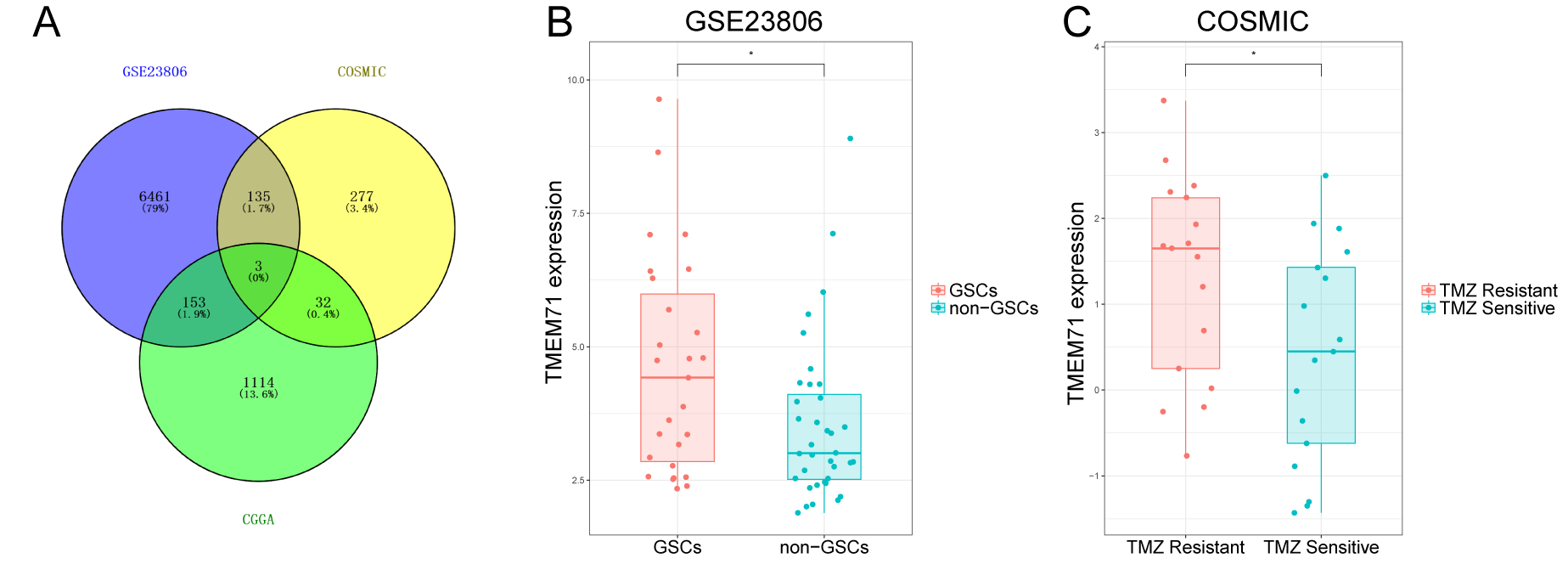

Supplement: Supplementary file 1 [file CNS-25-965-s001.tif]

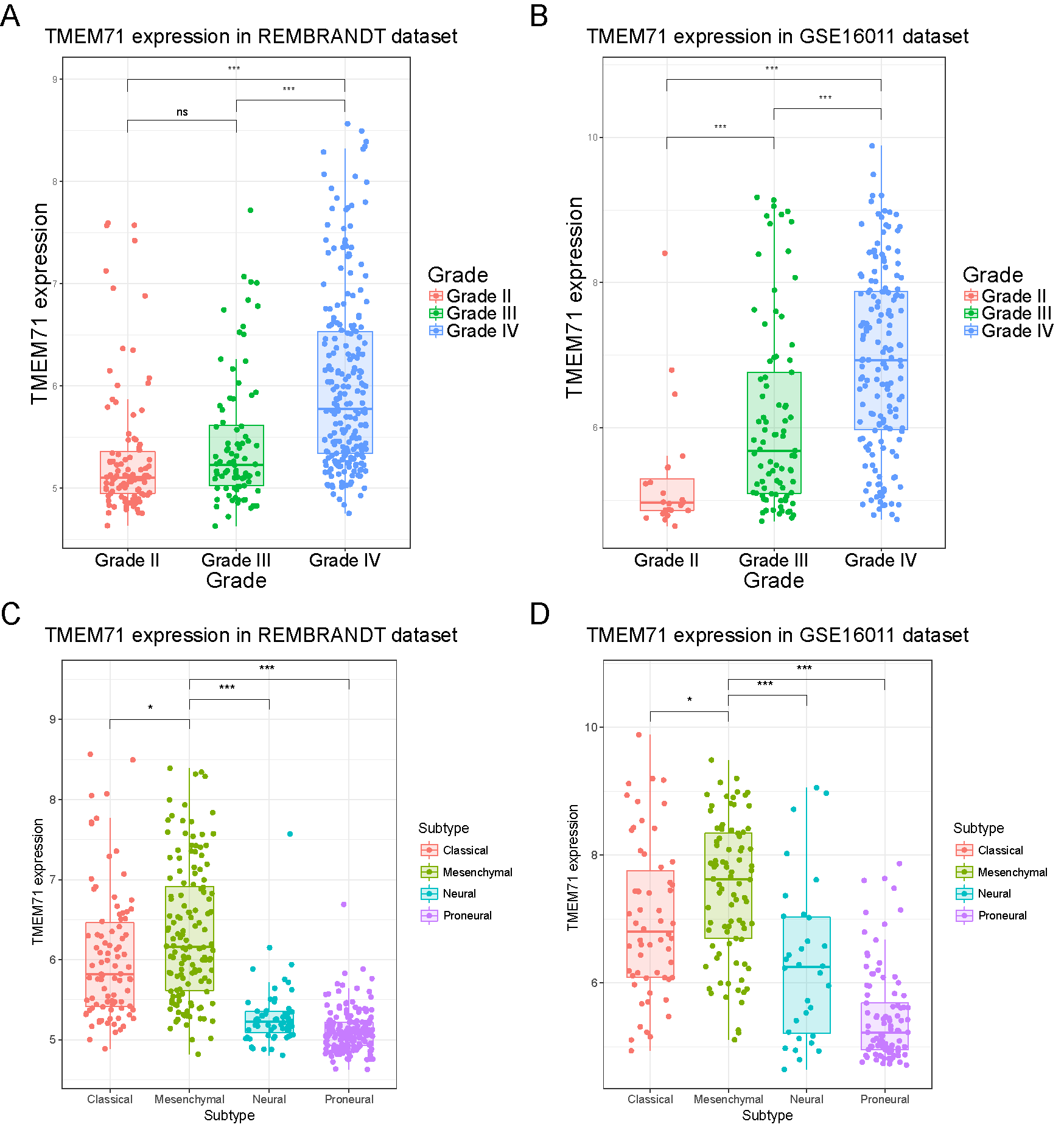

Supplement: Supplementary file 2 [file CNS-25-965-s002.tif]

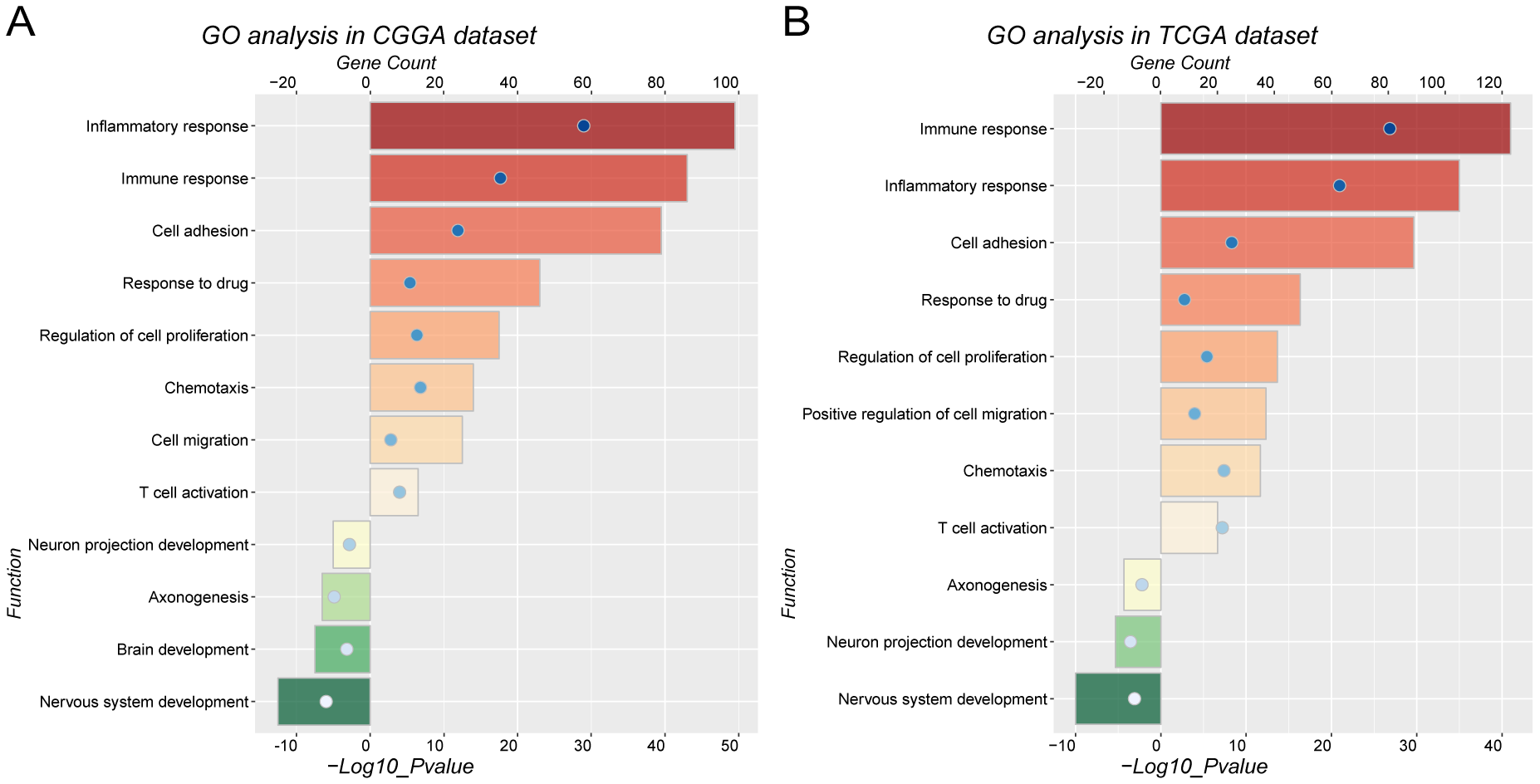

Supplement: Supplementary file 3 [file CNS-25-965-s003.tif]

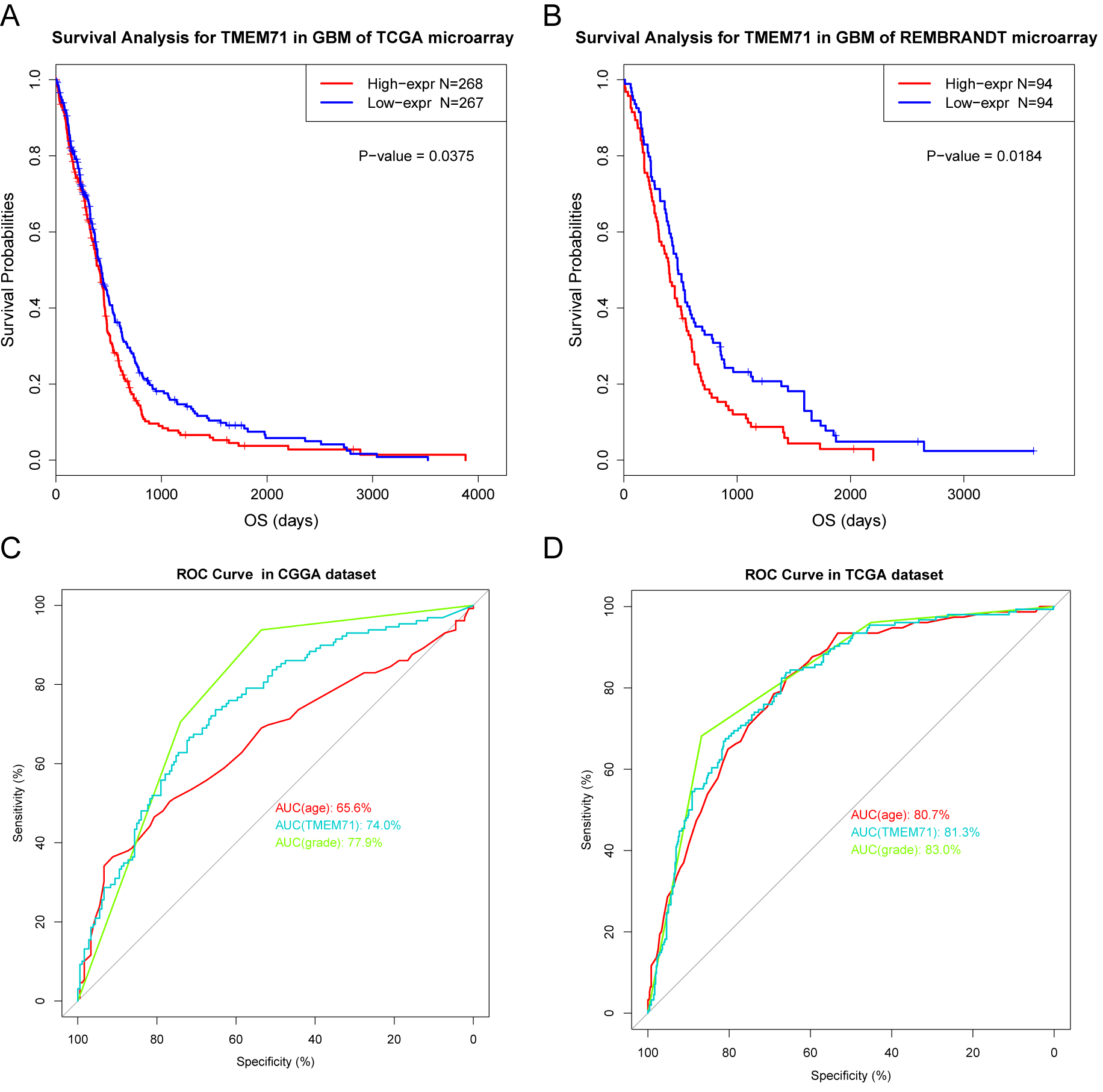

Supplement: Supplementary file 4 [file CNS-25-965-s004.tif]
