# Supplementary material for: Molecular and clinical characterization of TMEM71 expression at the transcriptional level in glioma
Source: CNS Neurosci Ther. 2019 Jun 10;25(9):965–75. doi: 10.1111/cns.13137 (PMC6698980; doi:10.1111/cns.13137)
Supplement: Supplementary file 5 [file CNS-25-965-s005.docx]

**Table S1. Univariate and multivariate analysis of OS in TCGA RNA sequencing database, GBM**

| **Variables** | **Univariate analysis** | | **Multivariate analysis** | | |
| --- | --- | --- | --- | --- | --- |
|  | **HR (95% CI)** | **p value** | **HR (95% CI)** | | **p value** |
| **TMEM71 expression** | 1.153 (1.009-1.317) | 0.036 | 1.285 (1.059-1.560) | 0.011 | |
| **Age at diagnosis** | 1.036 (1.018-1.053) | < 0.001 | 1.055 (1.028-1.084) | < 0.001 | |
| **Gender** | 0.875 (0.598-1.280) | 0.491 | - | - | |
| **TCGA subtype** | 1.027 (0.856-1.231) | 0.777 | - | - | |
| **IDH mutation status** | 0.263 (0.105-0.655) | 0.004 | 0.650 (0.145-2.920) | 0.575 | |
| **MGMT methylation** | 0.572 (0.356-0.919) | 0.021 | 0.658 (0.406-1.067) | 0.09 | |
| **KPS** | 0.990 (0.974-1.006) | 0.233 | - | - | |
