# Supplementary material for: Molecular and clinical characterization of TMEM71 expression at the transcriptional level in glioma
Source: CNS Neurosci Ther. 2019 Jun 10;25(9):965–75. doi: 10.1111/cns.13137 (PMC6698980; doi:10.1111/cns.13137)
Supplement: Supplementary file 6 [file CNS-25-965-s006.docx]

**Table S2. Univariate and multivariate analysis of OS in TCGA microarray database, GBM**

| **Variables** | **Univariate analysis** | | **Multivariate analysis** | | |
| --- | --- | --- | --- | --- | --- |
|  | **HR (95% CI)** | **p value** | **HR (95% CI)** | | **p value** |
| **TMEM71 expression** | 7.035 (1.571-31.494) | 0.011 | 18.43 (2.463-138.02) | 0.005 | |
| **Age at diagnosis** | 1.005 (0.988-1.022) | 0.569 |  |  | |
| **Gender** | 1.227 (0.795-1.893) | 0.355 |  |  | |
| **TCGA subtype** | 1.082 (0.900-1.301) | 0.403 |  |  | |
| **IDH1 mutation status** | 0.709 (0.416-1.206) | 0.204 |  |  | |
| **MGMT methylation** | 0.564 (0.364-0.872) | 0.01 | 0.921 (0.506-1.673) | 0.786 | |
| **Radiotherapy** | 0.412 (0.259-0.654) | < 0.001 | 0.498 (0.274-0.907) | 0.023 | |
| **Chemotherapy** | 0.336 (0.214-0.528) | < 0.001 | 0.442 (0.251-0.778) | 0.005 | |
| **KPS** | 0.970 (0.955-0.986) | < 0.001 | 0.961 (0.942-0.981) | < 0.001 | |
